# Genomic Signatures of Mitonuclear Coevolution in Mammals

**DOI:** 10.1093/molbev/msac233

**Published:** 2022-10-27

**Authors:** Ryan J Weaver, Samantha Rabinowitz, Kiley Thueson, Justin C Havird

**Affiliations:** Department of Ecology, Evolution, and Organismal Biology, Iowa State University, Ames, IA; Department of Natural Resource Ecology and Management, Iowa State University, Ames, IA; Department of Integrative Biology, University of Texas, Austin, TX; Department of Integrative Biology, University of Texas, Austin, TX; Department of Integrative Biology, University of Texas, Austin, TX

**Keywords:** coevolution, mitochondria, mitonuclear interactions, nuclear compensation

## Abstract

Mitochondrial (mt) and nuclear-encoded proteins are integrated in aerobic respiration, requiring co-functionality among gene products from fundamentally different genomes. Different evolutionary rates, inheritance mechanisms, and selection pressures set the stage for incompatibilities between interacting products of the two genomes. The mitonuclear coevolution hypothesis posits that incompatibilities may be avoided if evolution in one genome selects for complementary changes in interacting genes encoded by the other genome. Nuclear compensation, in which deleterious mtDNA changes are offset by compensatory nuclear changes, is often invoked as the primary mechanism for mitonuclear coevolution. Yet, direct evidence supporting nuclear compensation is rare. Here, we used data from 58 mammalian species representing eight orders to show strong correlations between evolutionary rates of mt and nuclear-encoded mt-targeted (N-mt) proteins, but not between mt and non-mt-targeted nuclear proteins, providing strong support for mitonuclear coevolution across mammals. N-mt genes with direct mt interactions also showed the strongest correlations. Although most N-mt genes had elevated *d*_N_/*d*_S_ ratios compared to mt genes (as predicted under nuclear compensation), N-mt sites in close contact with mt proteins were not overrepresented for signs of positive selection compared to noncontact N-mt sites (contrary to predictions of nuclear compensation). Furthermore, temporal patterns of N-mt and mt amino acid substitutions did not support predictions of nuclear compensation, even in positively selected, functionally important residues with direct mitonuclear contacts. Overall, our results strongly support mitonuclear coevolution across ∼170 million years of mammalian evolution but fail to support nuclear compensation as the major mode of mitonuclear coevolution.

## Introduction

The union of ancient prokaryotic lineages approximately 2 billion years ago (Williams and Embley 2014; Derelle et al. 2015) gave rise to the dual genomic nature of eukaryotes. This union established a hotspot for one of the most long-standing examples of molecular cooperation and conflict in biology—requisite interactions between two independently-evolving genomes within a single cell. Mitochondria are arenas for critical physiological pathways including cellular respiration, which requires coordinated interactions between mitochondrial-encoded (mt) proteins and nuclear-encoded, mitochondria-targeted (N-mt) proteins (Rand et al. 2004; Hill 2015).

Among the most intimate mitonuclear interactions are proteins in the respiratory complexes of the electron transport system (ETS), which perform oxidative phosphorylation (OXPHOS). In bilaterian animals, N-mt encoded OXPHOS subunits interact with mt protein subunits within four of the five ETS complexes. ETS complex II (CII; succinate dehydrogenase) is strictly nuclear encoded in bilaterian animals, providing an in situ negative control when examining mitonuclear interactions. Mitonuclear interactions are also required for the translation of the mt genome, where N-mt ribosomal proteins (N-mrps) are assembled on a scaffold of mt-encoded rRNAs and structural tRNAs to make up the mt ribosome (Bogenhagen et al. 2018). Other nuclear-encoded ribosomal proteins (cytoribo proteins) form the cytoplasmic ribosome, which is responsible for translating nuclear-encoded genes and provides a functionally analogous negative control for examining mitonuclear interactions in N-mrps. Other examples of mitonuclear interactions include the “charging” of mt-encoded tRNAs by nuclear-encoded aminoacyl-tRNA synthetase proteins (Adrion et al. 2016), small RNAs encoded by the mtDNA that interact with nuclear-encoded RNAs (Pozzi and Dowling 2022), and the replication, repair, and transcription of the mtDNA itself by nuclear-encoded proteins (Rand et al. 2004; Levin et al. 2014).

Given that mitonuclear interactions are precise, are ancient, and are critical for cellular energetics (Rand et al. 2004; Wolff et al. 2014; Van Der Sluis et al. 2015), maintaining their integrity may be a fundamental rule across eukaryotes. The mitonuclear coevolution hypothesis offers one solution for how these interactions remain functional. It posits that evolutionary change in one genome should exert selection for complementary changes in the other genome. In this way, mt and N-mt genes should be hotspots for molecular coevolution. A predicted genomic signature of molecular coevolution is a correlation between the evolutionary rates of interacting genes, known as evolutionary rate covariation (ERC; Clark et al. 2012; De Juan et al. 2013). Studies on the molecular evolution of mitonuclear genes in insects and bivalves support this prediction: mt and N-mt evolutionary rates are strongly and positively correlated, while non-mt interacting N genes show weak or no correlations with mt evolutionary rates (Yan et al. 2019; Piccinini et al. 2021). Similarly, in the plant genus *Silene*, species with fast mt evolution show fast evolution in N-mt genes, but not in control nuclear genes (Sloan et al. 2014; Havird et al. 2015, 2017). While mitonuclear coevolution has been supported in general across many systems (Gershoni et al. 2010; Burton et al. 2012; Levin et al. 2014; Mossman et al. 2019) mitonuclear ERC analyses have not been conducted in a vertebrate, despite the variation in mt evolutionary rates across this clade.

Mitonuclear coevolution could in theory arise from many mechanisms, but a specific mechanism termed the “nuclear compensation” hypothesis has received the most attention (Levin et al. 2014; Havird et al. 2017; Sloan et al. 2017; Iannello et al. 2019; Hill 2020). Under this hypothesis, mitonuclear coevolution occurs via nuclear compensation because mt genomes are prone to accumulate slightly deleterious mutations at a faster rate than N genomes (due to uniparental inheritance, lack of recombination, and effective haploidy; Lynch 1996; Lynch 1997; Lynch and Blanchard 1998; Neiman and Taylor 2009). Therefore, deleterious mt mutations should impose selection for compensatory changes in N-mt products to maintain mitonuclear coadaptation and functional mitochondria. Nuclear compensation may be especially important in vertebrate animals, as mt genomes typically accumulate mutations at 20 times the rate of the N genome, although significant variation exists across different lineages (Brown et al. 1979; Neiman and Taylor 2009; Lynch 2010; Allio et al. 2017). Other possible forms of mitonuclear coevolution include mt compensation, synergistic coevolution (where initially beneficial changes in one genome allow for new beneficial changes in the other genome), or coevolution driven by neutral changes (see fig. 1 in Sloan et al. 2017).

The most commonly tested prediction of nuclear compensation is that N-mt genes—but not mt or non mt-interacting N genes—should evolve under positive selection (Rand et al. 2004; Osada and Akashi 2012). The ratios of non-synonymous to synonymous substitution rates—that is *d_N_/d_S_*— is often used as a proxy for inferring positive selection. Elevated *d_N_/d_S_* ratios in N-mt compared to other N genes and mt-encoded genes have previously been used as evidence of nuclear compensation (Rand et al. 2004; Havird et al. 2015), although *d_N_/d_S_* ratios are similar between N-mt and mt genes in bivalves (Piccinini et al. 2021). Others have argued that increased *d_N_/d_S_* ratios in N-mt genes are due to relaxed, not positive selection, although this has been at least partially refuted in some taxa (Nabholz et al. 2013; Popadin et al. 2013; Havird and Sloan 2016; Havird et al. 2017). However, the nuclear compensation hypothesis also implies a temporal prediction that is rarely tested: N-mt amino acid substitutions should arise sequentially after mt substitutions if they are compensating for deleterious mt substitutions. Deducing this temporal pattern is complicated, but has been accomplished by mapping mt and N-mt amino acid substitutions onto phylogenies in concert with spatial analyses of resolved 3D structures of ETS complexes (Osada and Akashi 2012; Havird et al. 2015). Osada and Akashi (2012) noted that N-mt substitutions tend to follow mt substitutions in primate cytochrome C oxidase (ETS complex IV, CIV) and Havird et al. (2015) found that N-mt, but not mt, substitutions tended to occur at contact residues in ETS complexes, possibly increasing structural stability of the complexes. However, both studies suffered from a low number of substitutions, and it is unclear if such predicted patterns of nuclear compensation are widespread and robust across eukaryotes.

In this study we tested predictions of the mitonuclear coevolution hypothesis by analyzing mt and N genomes across ∼170 million years of mammalian evolution. We used publicly available sequence data to perform ERC analyses of amino acid substitution rates from mt genes, N-mt genes, and control N genes that do not interact with mitochondria. We predicted that if mt genomes and N-mt genes are coevolving, there should be relatively strong positive correlations in evolutionary rates between mt and N-mt genes. We also investigated *d_N_/d_S_* ratios, temporal patterns of substitutions, and spatial coordination of substitutions to directly test predictions of the nuclear compensation hypothesis. Specifically, under nuclear compensation N-mt genes should show the highest *d_N_/d_S_* ratios and N-mt substitutions should follow mt substitutions. Positively selected N-mt substitutions should also be overrepresented for direct contacts with mt-encoded residues.

## Results and Discussion

### Robust Signatures of Mitonuclear Coevolution in Mammals

**Fig. 1.**
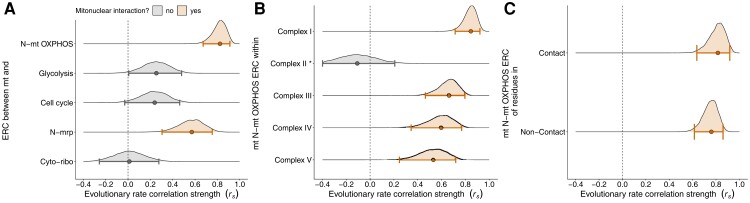
Evolutionary rate correlations among (*A*) mt genes and nuclear-encoded genes targeted to mitochondria (N-mt OXPHOS and mitoribosomal proteins), and non-mitochondrial targeted nuclear-encoded genes. The distribution of 10,000 bootstrap replicates of the correlation coefficient, *r_s_* are shown with dots showing the mean and bars showing the 95% confidence intervals (scatterplots of correlations are shown in supplementary fig. S1, 2, and 3, Supplementary Material online). (*B*) Within-complex ERC of mt and N-mt OXPHOS proteins. *CII correlation is between nuclear-encoded CII genes and all mt genes. All other correlations are between mt and N-mt genes within the mitonuclear ETS complex. (*C*) ERC of mt and N-mt OXPHOS genes that either contain or lack residues that directly contact mt residues. Nuclear genes targeted to ETS (N-mt OXPHOS); nuclear genes encoding mitoribosomal proteins (N-mrp); nuclear genes encoding cytosolic ribosomal proteins (Cyto-ribo).

Across the 58 mammals we investigated, we found that mt genes evolved faster than N-mt genes, ranging from ∼3.5 to ∼11.5 times faster, depending on the species. mt amino acid substitution rates also varied among species and were ∼3.3 times higher in the fastest species (Mongolian gerbil, *Meriones unguiculatus*) compared to the slowest species (platypus, *Ornithorhynchus anatinus*), which provided the variation needed to perform a robust ERC analysis. Overall, we found that evolutionary rates of mt and N-mt proteins were strongly positively correlated across 58 mammals, while non-mt interacting N protein evolution rates showed weak or no correlations with mt rates (fig. 1*A*). This supports a key prediction of the mitonuclear coevolution hypothesis and suggests that in lineages where evolutionary rates increase in one genome, rates also increase in interacting genes of the other genome as complementary mutations are selected to become fixed substitutions.

We normalized evolutionary rates of five nuclear-encoded gene sets (N-mt OXPHOS, N-mt ribosomal, glycolysis, cell cycle, and cytoplasmic-ribosomal genes) and mt rates to nuclear genomic rates of evolution based on a set of conserved mammalian orthologs. We then correlated mt rates to rates in each of the nuclear gene sets. Importantly, because of this normalization any correlations were not due to overall changes in molecular evolutionary rates owing to demographic features in particular lineages (e.g., changes in *N_e_*) and accounts for any mutation rate variation within lineages. After normalizing to background evolutionary rates, we found that mt and N-mt OXPHOS amino acid substitution rates were highly positively correlated (*r_s_* = 0.82, 95% CI = 0.67–0.91; fig. 1*A*; supplementary fig. S1, Supplementary Material online), while those of mt and N genes involved in a strictly N-encoded energy production pathway (glycolysis) were very weakly positively correlated (*r_s_* = 0.25, 95% CI = 0.001–0.47; fig. 1*A*). These patterns of ERC being highest between mt and N-mt proteins and lowest between mt and non-mt-interacting N proteins remain present after accounting for potential phylogenetic structure within our dataset (*e.g*., mt vs. Nmt: *r_s_* = 0.71, mt vs. glycolysis: *r_s_* = 0.19; supplementary table S2, Supplementary Material online). Bootstrapping analysis confirmed the differences in correlations were statistically robust, as the 95% confidence interval did not include a difference of zero (difference *r_s_* = 0.57, 95% CI = 0.33–0.84). Havird and Sloan (2016) used a similar approach across eukaryotes to show that N-mt OXPHOS genes, but not glycolysis genes, evolve faster in lineages with higher relative mt mutation rates (although see Piccinini et al. 2021).

We also found that evolutionary rates of mt genes and N-mt genes that encode mt ribosomal proteins were strongly correlated (N-mrp genes, *r_s_* = 0.57, 95% CI = 0.30–0.75; fig. 1*A*). Even though N-mrp proteins do not interact directly with the mt OXPHOS proteins examined here, we assume that mt OXPHOS rates are good predictors of evolutionary rates in mt rRNA genes. Our result is therefore likely indicative of mitonuclear coevolution in the mitoribosome machinery, as has been shown in other studies (Barreto and Burton 2013b; Sloan et al. 2014). For example, Yan et al. (2019) found that evolutionary rates of mt rRNA genes are highly correlated with those of N-mrp genes within insect lineages. In contrast to N-mrp genes, we found no correlation between mt rates and nuclear-encoded proteins that form the cytoplasmic ribosome (*r_s_* = 0.01, 95% CI = −0.26–0.27; fig. 1*A*). The difference in correlation between mt and N-mrp genes and mt and cytoplasmic ribosomal datasets was statistically robust (difference *r_s_* = 0.59, 95% CI = 0.16–0.90). As an overall negative control, we also investigated ERC between mt and nuclear-encoded cell cycle genes, which showed a weak positive correlation, similar to the glycolysis genes (*r_s_* = 0.24, 95% CI = −0.03–0.46; fig. 1*A*). While cell cycle proteins do not interact directly with mt gene products, mt processes are involved with and/or influenced by the cell cycle and therefore it is possible that these genes are physiologically linked (Arciuch et al. 2012; Xiong et al. 2012).

### Evolutionary Rate Correlations are Consistently High Across Mitonuclear ETS Complexes

Our results above show the stark differences in mitonuclear ERCs when nuclear proteins are targeted to mitochondria versus nuclear proteins not targeted to mitochondria. We can also make similar comparisons within OXPHOS complexes, as only nuclear proteins that closely interact with mt-encoded gene products should show high ERCs with mt DNA. As mentioned above, ETS complexes provide a way to test this prediction, as CII is entirely nuclear encoded in many eukaryotes, including mammals. We found that the rate correlation between CII genes and mt genes was weakly negative (fig. 1*B*; supplementary fig. S2, Supplementary Material online, *r_s_* = −0.11, 95% CI = −0.40–0.21), suggesting that CII is not co-evolving with mt genes, despite its role in electron transport and mt respiration. In contrast, we found strong ERCs in the other mitonuclear OXPHOS complexes with CI having a slightly higher rate correlation compared to CIV, CIII, and CV (fig. 1*B*; supplementary fig. S2, Supplementary Material online). CI and CIV have the most mt-encoded genes among the ETS complexes, suggesting mitonuclear interactions may be particularly critical in these complexes (Gershoni et al. 2010; Van Der Sluis et al. 2015). We note that CI and CIV also serve as the entry and exit points for electrons, respectively and could be experiencing stronger selection for coevolution due to the potential for increased free radical production (CI; Speijer 2019) and dysregulation of mt respiration (CIV; Arnold 2012; Pierron et al. 2012). When correlating evolutionary rates among proteins in different mt and nuclear ETS complexes, we generally found that mitonuclear correlations involving mt CI and CIV were high (supplementary fig. S3, Supplementary Material online), providing further support of the importance of mitonuclear coevolution in these complexes. However, differences in gene length between mt and N-mt components of the ETS complexes may impart a bias. Especially in complexes with relatively short mt components. For example, CIII has nine N-mt genes (∼1300 amino acids), yet only one mt-encoded protein (∼380 amino acids). However, evolutionary rates of CII proteins were slightly negatively correlated with proteins from all mt-encoded and nuclear-encoded ETS complexes (supplementary fig. S3, Supplementary Material online), showing that relative length of the dataset does not explain these trends.

Our results demonstrating the strong ERC of mitonuclear genes and mt genes both at the cellular and organellar-level are not unique to mammals. Our study of mitonuclear coevolution recapitulates the results of previous work: ERC analyses in bivalves showed strong correlations between mt and N-mt OXPHOS genes, except for those in CII (Piccinini et al. 2021) and in *Silene* species with fast-evolving mtDNA, N-mt OXPHOS evolution is also accelerated, except for CII genes (Havird et al. 2015). Our results are also supported by functional analyses in *Tigriopus* copepods that have inter-population divergence in mt genomes exceeding 15% but relatively low divergence across the entire N genome (Burton and Lee 1994; Willett and Burton 2004; Barreto et al. 2018). In this system, inter-population hybrids show reduced enzymatic capacity of mitonuclear OXPHOS complexes (Burton 1990; Ellison and Burton 2006; Barreto et al. 2015). However, CII activity is unaffected in mitonuclear mismatched hybrids (Ellison and Burton 2006), likely due to its strictly nuclear-encoded protein composition. These results all point to directly interacting mt and N-mt proteins showing the strongest signatures of mitonuclear coevolution. Moreover, mitonuclear coevolution has been supported in general across many systems (Gershoni et al. 2010; Burton et al. 2012; Levin et al. 2014; Sloan et al. 2017; Hill et al. 2019; Mossman et al. 2019).

### Spatial Proximity of N-mt OXPHOS and mt Proteins Weakly Influences Evolutionary Rate Correlations in Mammals

There is also a spatial component to mitonuclear coevolution. Those N-mt proteins that interact directly with mt gene products should show the strongest signatures of coevolution. Within the mitonuclear ETS complexes some amino acid residues are in close physical proximity to residues encoded by the other genome (contact sites). Previous studies have found some evidence that mitonuclear coevolution is particularly strong among mt and N-mt proteins that contain contact residues but weak among non-contact proteins/residues (Havird et al. 2015; Yan et al. 2019). Narrowing our N-mt OXPHOS dataset to only mitonuclear complexes (i.e., excluding CII) and partitioning them into “contact” or “non-contact” proteins (those with or without contact residues) revealed that mt ERC of contact proteins are somewhat stronger than non-contact proteins, but this difference is not statistically significant based on our bootstrap sampling (fig. 1*C*, difference in *r_s_* = 0.05, 95% CI = −0.08–0.17). Our results showing a lack of robust differences in ERCs between contact and non-contact proteins are consistent with ERC results from bivalves, where correlations were only slightly elevated for N-mt OXPHOS genes with direct contacts (Piccinini et al. 2021). However, ERCs of contact proteins in insects were much stronger than non-contact proteins (Yan et al. 2019). The importance of tight coevolution of N-mt genes with direct mt contacts may therefore vary across eukaryotes, although we note that the OXPHOS structures used to determine mitonuclear contacts in all these studies are based on mammalian species, suggesting our analysis of mammals may be based on the most relevant structural information.

### Evolutionary Rate Correlation Strength Varies Among Mammalian Lineages

There was also variation among mammalian lineages in our ERC results (supplementary fig. S1, Supplementary Material online). Primates, Cetartiodactyla, and Whippomorpha showed high evolutionary rate correlations between mt and N-mt OXPHOS genes (*r_s_* > 0.75) while Rodentia, Carnivora, and Chiroptera showed variable and weaker correlations (0.0 < *r_s_* < 0.46; supplementary fig. S1, Supplementary Material online). The evolution of flight in bats (Chiroptera) has been proposed to exert selection for mt efficiency, including signatures of positive selection on mt and N-mt OXPHOS genes (Shen et al. 2010). Our results provide little support for this pattern in bats, although only six species were included in our dataset. Our overall results were robust to removing primates from the data, as ERCs with N-mt OXPHOS proteins remained higher than those with glycolysis genes (*r_s_* = 0.66 vs. 0.48) and ERCs with N-mrp genes remained higher than those with cytoribo genes (*r_s_* = 0.22 vs. −0.11) and ERC with cell cycle genes remained low (*r_s_* = −0.08). Interestingly, although the overall correlation between mt and N-mt OXPHOS rates was similar for contact and non-contact proteins across all mammals, correlations within mammalian lineages were variable. Compared to contact genes, ERCs of non-contact N-mt OXPHOS genes were only weakly positive (Primates, Cetartiodactyla, 0.5 < *r_s_* < 0.65) or negative (Rodentia, Chiroptera, −0.71 < *r_s_* < −0.28; supplementary fig. S4, Supplementary Material online). Therefore, within lineages the spatial component of mitonuclear coevolution is more strongly supported.

Overall, our results strongly support mitonuclear coevolution across mammals. Confirming our overall results, an unpublished thesis became available while this study was in review that also shows strong ERCs between mt and N-mt OXPHOS proteins (Dhawanjewar 2022). Previous studies in diverse lineages have also suggested mitonuclear coevolution may be a rule for eukaryotes (see Rand et al. 2004; Sloan et al. 2018; Hill et al. 2019 for reviews). If so, this implies that mt and N-mt genes are coadapted within an evolutionary lineage and breaking up coevolved mitonuclear genotypes during hybridization may produce offspring with compromised mt functions and low fitness. Accordingly, some of the most compelling evidence for mitonuclear coevolution comes from crossing studies where hybrids with mismatched mitonuclear genotypes show decreased fitness (Ellison and Burton 2008; Barreto and Burton 2013a; Sloan et al. 2017). Given the implications of mitonuclear coevolution for speciation and other evolutionary processes (Toews and Brelsford 2012; Bar-Yaacov et al. 2015; Barreto et al. 2015; Hill 2016; Sloan et al. 2017; Connallon et al. 2018; Hill et al. 2019), it is important to determine when—in an evolutionary context—and where—both at the species level and the protein level—selection for mitonuclear coevolution is strongest. For example, lineages with lower energetic requirements (Shen et al. 2010) or those with accessory N-encoded OXPHOS proteins (Weaver 2019; Weaver et al. 2020) may be under less stringent selection to maintain mitonuclear interactions. ERC analyses offer a promising approach to identify these patterns, but have so far been applied sparingly to test for mitonuclear coevolution. One weakness of this approach may be the timescales required to generate the variation in evolutionary rates needed to perform ERC. To date, ERC analyses have been conducted on only deeply divergent animal lineages spanning hundreds of millions of years of evolution. An open question is whether mitonuclear coevolution can be detected on shorter timescales using ERC. Parallel co-evolutionary dynamics also play out in plants, where plastid-nuclear interactions have been shown to drive ERC patterns (Williams et al. 2019). ERC analyses could also be applied to investigate which nuclear genes are coevolving with younger endosymbionts (Bennett and Moran 2015).

### Mitonuclear d_N_/d_S_ Ratios Provide Weak Support for Nuclear Compensation in Mammals

**Fig. 2.**
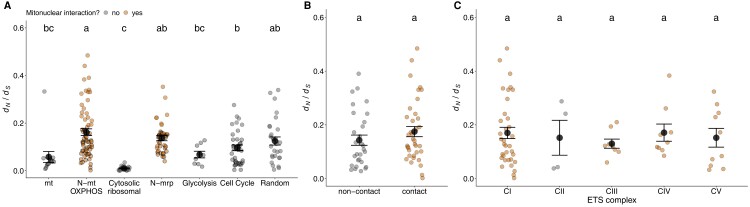
*d*_N_ */d*_S_ ratios of mitochondrial and nuclear gene sets. (*A*) Comparisons of *d*_N_*/d*_S_ ratios of mt, nuclear-encoded mitochondrial-interacting (N-mt OXPHOS and N-mrp) and nuclear-encoded, non-mitochondrial-interacting genes. (*B*) *d*_N_*/d*_S_ ratios of N-mt OXPHOS genes that either contain or lack residues that directly contact mt residues. (*C*) Within-complex *d_N_/d_S_* ratios of N-mt OXPHOS genes. Small dots represent individual genes, larger dark circle and error bars show the mean and standard error, respectively. Within each panel, the mean *d*_N_*/d*_S_ ratio of groups that do not share a letter are considered statistically significant from ANOVAs after Tukey HSD correction for multiple comparisons.

Nuclear compensation is often assumed to be the dominant mechanism of mitonuclear coevolution, given that mt genomes are prone to accumulate slightly deleterious mutations according to classic theory (Neiman and Taylor 2009). However, few studies have explicitly tested predictions that would provide critical support for nuclear compensation over other forms of mitonuclear coevolution. One such prediction is a directionality for positive selection: N-mt genes, but not mt genes, should be under intensified positive selection to fix compensatory changes. While purifying selection is likely the main type of selection acting on mt and N-mt genes across most eukaryotes, a relative increase in *d*_N_*/d*_S_ could indicate intensified positive selection in N-mt compared to noninteracting N genes or mt-encoded genes. Here, we found increased *d*_N_*/d*_S_ ratios in N-mt OXPHOS and N-mt ribosomal genes compared to the control glycolysis (Tukey HSD difference = 0.09, *P* = 0.035) and cytoplasmic ribosomal gene sets (difference = 0.13, *P* < 0.0001, although *d*_N_*/d*_S_ ratios were always < 1 in our analyses, fig. 2*A*). *d*_N_*/d*_S_ ratios in N-mt OXPHOS genes were also higher than in mt-encoded genes (difference = 0.10, *P* = 0.002). This supports the results of previous studies (Nabholz et al. 2013; Havird and Sloan 2016) and is consistent with nuclear compensation driving mitonuclear coevolution, as elevated *d*_N_*/d*_S_ ratios can be a signature of positive selection.

However, elevated *d*_N_*/d*_S_ ratios can also be caused by relaxed selection. There are compelling reasons why N-mt OXPHOS and N-mt ribosomal genes could be evolving under relatively relaxed selection. First, in contrast to mt proteins, which make up the catalytic core of ETS complexes, N-mt OXPHOS genes often occupy peripheral positions (Fiedorczuk et al. 2016; Zong et al. 2018) and may play less central roles in electron transport than mt genes, but rather act as environment-dependent regulators of the complex (Čunatova et al. 2020). Supporting this, we identified core (18%) versus peripheral (82%) N-mt OXPHOS genes and found higher *d*_N_*/d*_S_ ratios in the peripheral N-mt genes (core = 0.06, peripheral = 0.18). Similarly, N-mt ribosomal proteins participate in translating only 13 genes in mammals compared to ∼20,000 genes for N cytoplasmic-ribosomal genes. Sloan et al. (2014) used the *Silene* system to show that elevated *d*_N_*/d*_S_ ratios in N-mt ribosomal genes are likely caused by both positive and relaxed selection.

Additionally, comparing *d*_N_*/d*_S_ ratios across different genomes with radically different mutation rates may not be appropriate. Comparing relevant sets of genes within the same genome (subject to similar mutation rates) may be more applicable. Here, we found that *d*_N_*/d*_S_ ratios of contact N-mt OXPHOS genes were only slightly, and not statistically significantly, elevated compared to non-contact N-mt OXPHOS genes (difference = 0.03, *P* = 0.225, fig. 2*B*). Similarly, when examining N-mt OXPHOS contact genes only, there was a slight trend, though statistically non-significant, for genes with a higher proportion of mt contact residues to have higher *d*_N_*/d*_S_ ratios (supplementary fig. S5, Supplementary Material online, increase of 0.01 in *d*_N_*/d*_S_ per 10% increase in contact residues, *r* = 0.184, *P* = 0.249). As all mt-encoded proteins contact nuclear proteins, this analysis is impossible in mt genes, making it unclear if this pattern is specifically due to nuclear compensation or another form of mitonuclear coevolution. The other control nuclear gene sets (cell cycle and random orthologs) showed relatively high *d*_N_*/d*_S_ ratios, calling into question whether *d*_N_*/d*_S_ ratios are truly elevated in N-mt genes compared to sets of genes in the nuclear genome that are likely not evolving under positive selection. Moreover, *d*_N_*/d*_S_ ratios were similar across N-mt OXPHOS genes for all ETS complexes (fig. 2*C*), including CII. However, only four CII genes are present in mammals, making statistical inferences difficult. Under nuclear compensation, CII genes should have relatively low *d*_N_*/d*_S_ ratios as they do interact directly with mt proteins and should not be under positive selection. Taken together, while *d*_N_*/d*_S_ ratios are largely as predicted under nuclear compensation, multiple alternative explanations may explain their distributions and not all predictions of nuclear compensation involving *d*_N_*/d*_S_ ratios are supported.

### Spatiotemporal Analyses Do not Support Nuclear Compensation Within Mammalian ETS Complex IV

Implicit to the nuclear compensation hypothesis is a temporal pattern of slightly deleterious mt substitutions occurring first, followed by the fixation of N-mt mutations that compensate for the deleterious impact (Rand et al. 2004). Moreover, compensatory N-mt substitutions should be over-represented in proteins and at amino acid residues that physically contact mt residues within ETS complexes. A notable example of the spatiotemporal pattern of nuclear compensation was found in a landmark study examining five primates. Osada and Akashi (2012) found that substitutions between interacting pairs of amino acids in CIV tended to occur on the same phylogenetic branch more often for mt/N-mt pairs than pairs within the same genome (e.g., mt/mt or N-mt/N-mt pairs). Unfortunately, the occurrence of mt and N-mt changes on the same branch precludes determining the predicted temporal sequence of mt changes occurring first. They also found that mt changes tended to precede nuclear changes for pairs under positive selection. However, among the five primates in their study, only four candidate sites under positive selection were found and at two of those sites the substitutions occurred on the same phylogenetic branch.

**Fig. 3.**
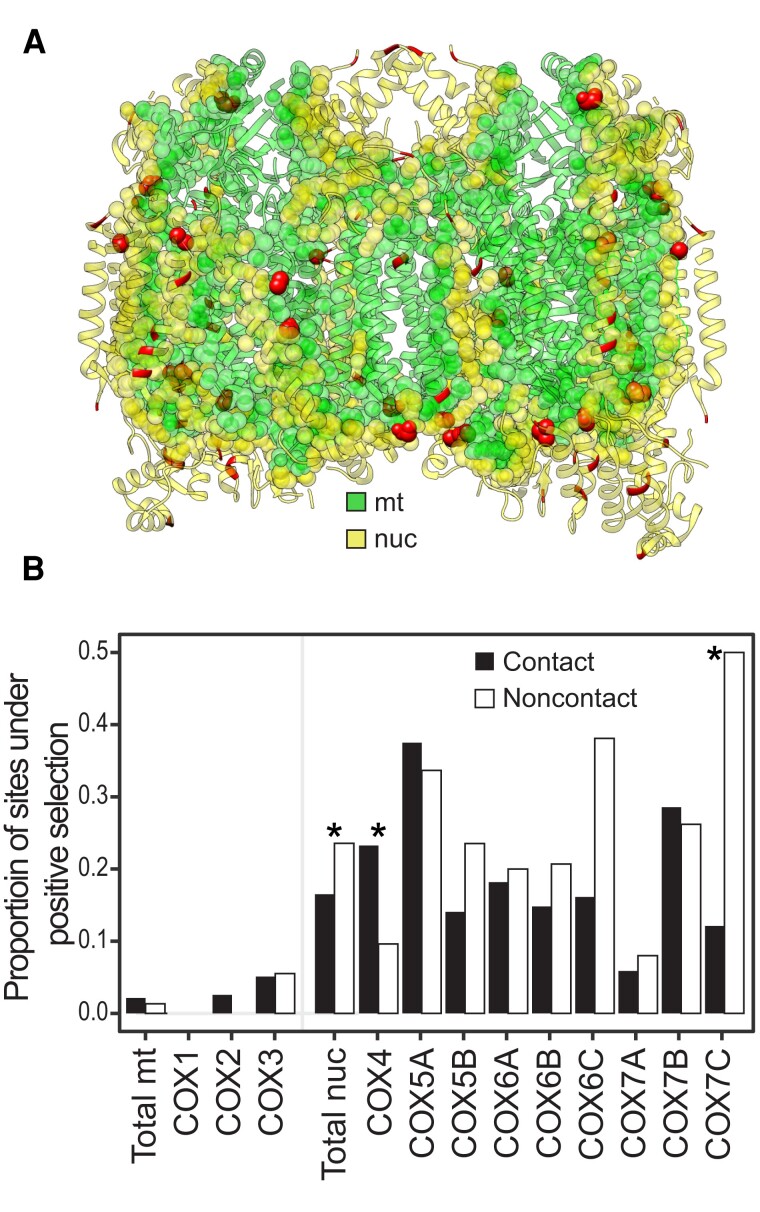
Spatial context of mitonuclear changes within ETS Complex IV. (*A*) CIV structure (based on Tsukihara et al. 2003) with mitochondrial residues in green, nuclear residues in yellow, contact residues as spheres, and non-contact residues as ribbons. Residues identified as evolving under positive selection in any analysis are shown as red spheres. (*B*) Estimates of the proportion of mitochondrial and nuclear amino acid sites under positive selection within ETS Complex IV. Asterisks denote a significant difference between contact and non-contact residues of the gene or genome determined by a Fisher's exact test. Analyses of residues that passed a more stringent test of positive selection are presented in supplementary figure S6, Supplementary Material online.

Here, we performed a similar analysis on CIV across mammals to investigate whether the spatiotemporal mode of substitutions supports nuclear compensation. We focused on CIV as an example given its previous use (Osada and Akashi 2012), the high ERC in CIV (fig. 1*B*), and high *d*_N_*/d*_S_ ratios in N-mt CIV genes (fig. 2*C*). However, we emphasize that this method can and should be used for other ETS complexes, other mitonuclear protein complexes, and in other taxa. We first found that sites under positive selection were ∼10 times more frequent in N-mt compared to mt genes (∼20% vs. 2% of sites, fig. 3; supplementary fig. S6, Supplementary Material online), supporting the prediction that higher *d*_N_*/d*_S_ in N-mt compared to mt genes is due to positive selection. While this is consistent with nuclear compensation, a more specific prediction is that N-mt sites that contact mt sites should especially be under positive selection compared to non-contact sites. We did find this pattern in one gene when examining N-mt genes within CIV individually (*COX*4 contact sites were ∼2.4 times more likely to be under positive selection, *P* = 0.036, Fisher's exact test, fig. 3). However, when summing across all N-mt CIV genes, the opposite pattern was found. Sites lacking mt contacts were ∼1.4 times more likely to be under positive selection than contact sites (*P* = 0.022, Fisher's exact test, fig. 3). Therefore, the spatial component of the nuclear compensation hypothesis was not supported by our site-specific CIV analyses in mammals. A more stringent test to identify sites under positive selection gave similar results (supplementary fig. S6, Supplementary Material online). Although a low number of positively selected sites in mt CIV genes prevented any meaningful comparisons, under nuclear compensation positively selected mt sites should not be enriched for nuclear contacts, while under mt compensation mt sites–but not N-mt contact sites–are predicted to be under positive selection.

**Fig. 4.**
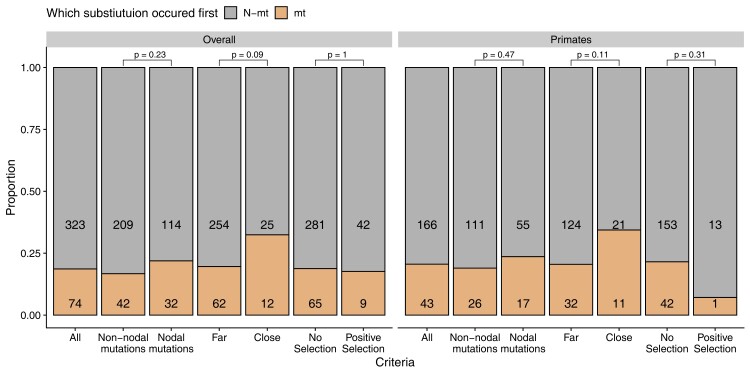
A summary of the relative timing of mt and nuclear (N-mt) amino acid substitutions within ETS complex IV genes that are near each other (contact pairs). Numbers within each bar show the count occurrences when N-mt (above) and mt (below) changes occurred sequentially first within an mt-N-mt contact pair. The complete dataset of CIV contact pairs (Overall, All) and a dataset that considered only primates (Primates, All) was filtered to select for sites that are more likely to show evidence of nuclear compensation: 1) whether the substitutions occurred within two branches along the root to tip node path of the phylogenetic tree (Close vs. Far), whether the N-mt gene showed evidence of positive selection, and whether it was a functional nodal mutation (see main text for details). Fisher's exact tests were used to evaluate whether the mt changes occurred first more often in Close versus Far comparisons, in genes under positive selection versus not under selection, and in functional nodal mutations versus not.

We also found no support for a temporal substitution pattern consistent with nuclear compensation in mammalian CIV. Both across all mammals included in this study and when considering only primates, the majority of mt substitutions did not occur sequentially before N-mt substitutions within contact site pairs (fig. 4). We identified 397 pairs of mt/N-mt contact sites with amino acid substitutions somewhere in the mammalian phylogeny. In only 74 (18.6%) of those instances, mt substitutions preceded N-mt substitutions, while the majority showed the opposite pattern (323 or 81.4%; fig. 4). N-mt substitutions actually preceded mt substitutions *more* often than expected by chance (χ^2^ = 156.17, *P* < 0.0001, fig. 4). A similar pattern was found within the primate lineages: in 79.4% of cases N-mt substitutions occurred first (χ^2^ = 72.4, *P* < 0.0001, fig. 4).

N-mt sites that are evolving under neutral processes may obscure these temporal results, as nuclear compensation predicts N-mt substitutions should follow mt substitutions due to positive selection for compensation (Rand et al. 2004). Therefore, we restricted our analyses to only contact sites where N-mt residues were determined to be under positive selection. Across all mammals, N-mt changes under positive selection still tended to precede mt changes at interacting sites (χ^2^ = 5.3, *P* = 0.021, fig. 4). N-mt contact sites found to be under positive selection were about equally as likely to have N-mt changes occur first compared with N-mt sites not under positive selection (fisher's exact test, odds ratio = 0.93, 95% CI = 0.38–2.05, *P* = 1; fig. 4). Within primates, N-mt changes under positive selection still preceded mt changes most of the time (odds ratio = 0.28, 95% CI = 0.006–1.98, *P* = 0.31). Similar to the analyses considering all mammals, amino acid changes at N-mt sites occurred before mt substitutions more frequently in primates regardless of whether the site was under positive selection (fig. 4).

We also restricted our analyses to substitutions that occurred somewhat close in tree space, as compensating nuclear changes may go to fixation relatively quickly following a deleterious mt substitution. However, a similar pattern in opposition of nuclear compensation was found when considering “close” versus “far” substitutions: despite more instances of N-mt changes preceding mt changes in all cases, mt substitutions were about twice as likely to occur before N-mt changes when they occurred close in tree space versus far across the entire phylogeny (odds ratio = 1.96, 95% CI = 0.85–4.3, *P* = 0.09; fig. 4) and when analyses were confined to primates (odds ratio = 2.02, 95% CI = 0.79–4.9, *P* = 0.11; fig. 4). In our most stringent analysis, we investigated only close substitutions with N-mt sites under positive selection. We found only one site pair that met these criteria in the overall dataset and none when we limited the analyses to primates.

Finally, we restricted our analyses to only those substitutions that were predicted to have functional consequences to the protein. Briefly, we identified functional nodal mutations as described by Levin and Mishmar (2017) based on predictive models that use frequencies of amino acid changes within a collection of homologous genes and 3D structures of ETS proteins to putatively identify deleterious changes. Still, in every case of different criteria we applied when considering functional nodal mutations, N-mt substitutions occurred before mt substitutions (fig. 4). Compared to a dataset that included mutations putatively tolerated, nodal mutations were slightly–yet non-significantly–more likely to occur before N-mt substitutions (all taxa: odds ratio = 1.4, 95% CI = 0.81–2.4, *P* = 0.23; primates only: odds ratio = 1.32, 95% CI = 0.62–2.8, *P* = 0.47; fig. 4; supplementary table S3, Supplementary Material online). However, in none of the analyses above did mt substitutions precede N-mt substitutions more than 50% of the time, leading us to conclude that our results fail to support the temporal predictions of nuclear compensation.

**Fig. 5.**
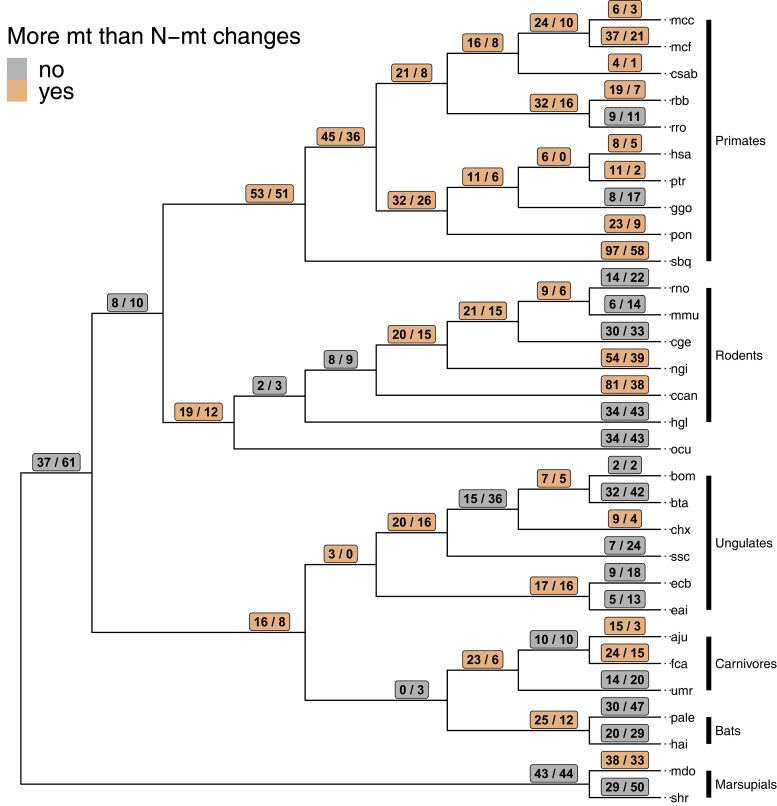
The estimated number of mt (left) and nuclear (N-mt, right) amino acid changes in ETS complex IV along each branch of our mammalian phylogeny. Orange shading indicates branches with more mt than N-mt substitutions, while gray shading shows the opposite. Tip labels correspond to KEGG codes for species names.

A potentially interesting pattern emerged when we mapped the number of mt and N-mt amino acid changes along each branch of the mammal phylogeny without consideration of whether they were contact residues (fig. 5). After the ancestral node leading to all primates in the phylogeny, all but two of the 18 branches have more mt amino acid changes than N-mt changes. Conversely, all other lineages (e.g., rodents, ungulates, carnivores) show the opposite pattern: more N-mt than mt substitutions on most branches of the phylogeny (20 of 37). Combined with our ERC results that show a stronger correlation between mt and N-mt evolutionary rates in primate OXPHOS genes, this suggests that mitonuclear coevolution may be stronger in primates than other mammals due to accelerated mt evolution in this group. Previous work has invoked the brain-energy hypothesis (Grossman et al. 2004) to suggest that nuclear compensation should be most prominent in the primates if it is the dominant form of mitonuclear coevolution (Osada and Akashi 2012). However, we found no support for nuclear compensation when limiting our spatiotemporal analyses to primates, as N-mt changes tended to occur first in all analyses (fig. 4).

Overall, the spatiotemporal pattern of molecular evolution in our study does not support nuclear compensation as the mechanism of mitonuclear coevolution in CIV of mammals. This may be due to technical limitations of our study. First, dividing our analyses into contact and non-contact residues (within a 5-angstrom window) is likely an oversimplification, as some amino acid substitutions may have effects outside of their immediate spatial vicinity (Anishchenko et al. 2017). However, other studies have used larger windows (10-angstrom) yet found weak support for the predicted temporal substitution pattern (Osada and Akashi 2012). Second, we identified contact sites using a single 3D structure based on the CIV complex of cow (*Bos taurus*) and there may be some differences in the structure of CIV among other mammals –potentially affecting which sites are classified as contact or noncontact. Third, whether mitonuclear coevolution is driven by a few critical changes in interacting residues (Meiklejohn et al. 2013; Moran et al. 2021) or many accumulated changes across large numbers of mitonuclear genes (Pereira et al. 2016; Barreto et al. 2018) is an open question, and our analyses would likely only detect the latter scenario. Fourth, our phylogenetic sampling of mammals may be too widespread to detect coordinated changes in interacting genes, which may occur over a relatively short time span. Osada and Akashi (2012) identified changes occurring on the same branch of the primate phylogeny as possible evidence of nuclear compensation. While this prevents testing a key temporal prediction of nuclear compensation, it may be a more reasonable timescale to detect changes relevant to mitonuclear coevolution. However, others have suggested sites showing covarying patterns of substitutions due to coevolution may be evolving slowly, meaning that such patterns can only be detected across deep phylogenies (Talavera et al. 2015), although this was based on nuclear-nuclear interacting proteins. Future studies using a denser sampling of species across multiple timescales and protein complexes should be encouraged.

Technical limitations of our study notwithstanding, it is possible that nuclear compensation plays a minor role in shaping patterns of mitonuclear coevolution. Nuclear compensation is often assumed to dominate across eukaryotes because mt genomes are predicted to accumulate deleterious mutations based on classic evolutionary theory (Hill and Robertson 1966; Lynch et al. 2006; Neiman and Taylor 2009). However, recent modeling studies have suggested mt genomes may not accumulate mutations as predicted for non-recombining genomes due to selection during transmission bottlenecks and other features of mtDNA heredity (Christie and Beekman 2017; Edwards et al. 2021). Some empirical evidence supports this in humans and *Drosophila* (Cooper et al. 2015). Other forms of coevolution in mitonuclear protein complexes include intragenic compensation—mito-mito or Nmt-Nmt—which has been supported in some studies (Kern and Kondrashov 2004; Breen et al. 2012; Christy et al. 2017; Levin and Mishmar 2017; Wernick et al. 2019).

Other forms of mitonuclear coevolution are also possible, including initial changes in either genome being neutral or beneficial followed by synergistic changes in the other genome (Sloan et al. 2017). An important consideration in disentangling different forms of mitonuclear coevolution is determining whether changes are deleterious or beneficial. Such studies are difficult, but not impossible. For example, Rand and colleagues have used factorial backcrossing designs to examine the fitness of individual nuclear and mt genotypes, as well as their interactions (Montooth et al. 2010; Mossman et al. 2016; Rand et al. 2021). The fitness of mitonuclear genotypes is also dependent on the environment (G × G × E interactions; Rand et al. 2018; Hill et al. 2019; Mossman et al. 2019). It may therefore be challenging to determine whether initial mt changes are indeed deleterious, as predicted under nuclear compensation, outside of evaluating a few well-characterized mutations of consequence in model organisms (Meiklejohn et al. 2013; Moran et al. 2021).

Levin and Mishmar (2017) combined predictive models that use frequencies of amino acid changes within a collection of homologous genes and 3D structures of ETS proteins to putatively identify deleterious changes, so called functional nodal mutations. Here, we adapted those methods to predict the functional consequence of mt amino acid changes on protein function or stability in complex IV. Still, those nodal mutations of mt-encoded amino acids mostly occurred after amino acid changes of interacting N-mt residues.

## Conclusions

Given their dual genomic nature, maintaining functional mitochondria through mitonuclear coevolution may be a rule for eukaryotes. Here, ERC analyses revealed a clear pattern of mitonuclear coevolution across ∼170 million years of mammalian evolution. While the strength of this pattern varied across taxonomic lineages and OXPHOS complexes, coordinated rates of evolution between interacting mitonuclear proteins were strongly supported. However, the specific form of mitonuclear coevolution known as nuclear compensation was not supported through spatiotemporal analyses of amino acid substitutions in mammalian CIV. Specific predictions that can distinguish nuclear compensation from other types of mitonuclear coevolution are often not explicitly tested and even general patterns of nuclear compensation based on *d*_N_*/d*_S_ ratios are not found in all lineages (Piccinini et al. 2021). We suggest nuclear compensation may be important in some eukaryotic lineages (e.g., those experiencing sudden increases in mt mutation rates, Havird et al. 2015; Havird et al. 2017), but should not be assumed to be the default form of mitonuclear coevolution across eukaryotes.

## Materials and Methods

### mt and Nuclear Gene Retrieval

For each of the 58 mammal species and *Alligator mississppiensis*, we retrieved amino acid sequences using custom scripts for the 13 mt OXPHOS genes, 74 N-mt OXPHOS genes, 40 mitoribosomal proteins genes (N-mrp), 20 cytoplasmic ribosomal genes (cytoribo), 13 cell cycle genes, and 9 glycolysis genes from the Kytoto Encyclopedia of Genes and Genomes (KEGG; Kanehisa and Goto 2000; Kanehisa et al. 2006) and NCBI's GenBank (supplementary table S1, Supplementary Material online). We also selected 30 orthologous nuclear genes at random using OrthoMam (Scornavacca et al. 2019) and retrieved protein sequences from 50 of the target species. Random orthologs from the remaining nine species’ protein sequences were retrieved from the REFSEQ protein database on NCBI.

### Data Cleaning and Alignment

Nuclear-encoded gene products that function in mitochondria contain a mt-targeting sequence motif at the N-terminus, which is cleaved upon mt entry and does not function in mitonuclear interactions. To avoid bias of elevated evolution rates in these sections of the N-mt OXPHOS and N-mrp genes, we trimmed the sequences after identifying the targeting motifs by using TargetP (Armenteros et al. 2019). We aligned the sequences for each gene using MUSCLE (Edgar 2004) implemented in MEGA X (Kumar et al. 2018) then trimmed all alignments using TRIMAL (Capella-Gutiérrez et al. 2009). We manually inspected each alignment to identify and remove paralogs and/or isoform variants. For each gene set, we concatenated the alignments using Sequence Matrix (Vaidya et al. 2011).

### Phylogenetic Analysis and Evolutionary Rate Calculations

We estimated the evolutionary rates of each set of concatenated amino acid sequence alignments (supplementary table S1, Supplementary Material online) by estimating branch lengths on an amino acid phylogeny inferred via maximum likelihood using RAxML (Stamatakis 2014). We constrained the starting tree topology to a consensus tree made from a sample of 1000 trees containing our focal taxa from vertlife.org (Upham et al. 2019). In other words, the topology was fixed to represent known mammalian species relationships, not estimated based on input sequence data. We used *Alligator mississippiensis* as the outgroup in phylogenetic analyses but did not include it in ERC analyses. Branch lengths were estimated using the best-fitting amino acid substitution model with a gamma distribution (-m PROTGAMMAAUTO) using 1000 rapid bootstraps followed by ML search and optimization. In the case of the concatenated mt gene set, the parameters were the same except the substitution model was set to -m PROTGAMMAIMTART, which is specifically designed for mt data. We extracted root-to-tip branch lengths using the distRoot function in “adephylo” and “ape” packages in R (Paradis and Schliep 2019). We also estimated branch lengths for phylogenies generated for each OXPHOS complex individually based on concatenated sets of mt and N-mt genes.

### ERC Analyses

A potential confounding effect of ERC analyses is that demographic factors may produce autocorrelation among gene sets that are not coevolving. For example, an observed correlation between mt and N-mt OXPHOS evolutionary rates may reflect different effective population sizes among species. To control for this, we normalized each target gene set to the background evolutionary rate by dividing the branch lengths of the target gene set by the branch lengths obtained when using a subset of half of the random nuclear orthologs. We haphazardly split the random nuclear ortholog genes into two groups (Nrand1 and Nrand2) before concatenation and performed phylogenetic analyses on them separately. To avoid auto correlation, we then used branch lengths of the Nrand1 group to normalize the mt gene set and the Nrand2 group to normalize the target N gene sets. In this way, we normalized our data to background evolutionary rates in a species while avoiding autocorrelation from normalizing to the same set of genes.

To estimate evolutionary rate correlations, we conducted spearman rank correlation tests on the normalized branch lengths of mt-encoded OXPHOS genes and each of the five nuclear-encoded gene sets, ETS complex gene sets, and contact and non-contact subsets (supplementary table S1, Supplementary Material online) using *corr.test* in R (version 4.0.0 R Core Team 2017). We also used a phylogenetic correlation test using *phylo.cor.test* in R to test for ERCs of mt and target N gene sets while accounting for shared ancestry of taxa. To statistically test for differences in correlations within our gene sets, we bootstrapped the resulting spearman rank correlation estimates, *r_s_,* for each group of 10,000 iterations using the *boot* function in R. We took the difference of the *r_s_* distribution of mt from each nuclear gene dataset *r_s_* distribution then calculated the 95% confidence intervals from the resulting *r_s_* distribution using the *boot.ci* function in R with type = bca. When the mean difference in *r_s_*and 95% confidence intervals do not include zero we interpreted that comparison to be statistically robust (at *P* = 0.05, more stringent thresholds to account for multiple testing produced similar results).

### Estimating d_N_/d_S_Values

We estimated a single *d*_N_*/d*_S_ value (model = 0, NSsites = 0) from nucleotide sequence alignments of individual genes (supplementary table S1, Supplementary Material online) using codeml in PAML 4.9j (Yang 2007). We tested for differences in *d_N_/d_S_* among datasets using ANOVA with a Tukey HSD post-hoc correction for multiple testing in R.

### Evaluating Nuclear Compensation based on Spatiotemporal Patterns of CIV Substitutions

We evaluated spatiotemporal patterns of amino acid substitutions to test predictions of the nuclear compensation hypothesis. We focused on cytochrome c oxidase (CIV), given previous results in primates (Osada and Akashi 2012), evidence of strong mitonuclear coevolution based on our ERC analyses (fig. 1*B*), and data availability. We also used a subset of 30 mammalian species given data availability (fig. 5; supplementary fig. S7, Supplementary Material online).

Using the mt and N-mt CIV datasets, we retained instances of only non-synonymous substitutions. We restricted our analysis to pairs of mt- and nuclear-encoded residues that physically contacted each other (i.e., contact residues). To identify contact residues, we used a similar method as Forsythe et al (2019). Briefly, a known mammalian CIV protein structure model (PDB: 1V54; Tsukihara et al. 2003) was imported into Chimera v1.12 (Petterson et al. 2004) and mitonuclear contact sites were identified using the “identify contacts/clashes” tool with default parameters except that overlap was set to ≤ −1 Å to increase sensitivity. These parameters return all mt:N-mt contact pairs within a 5 Å window. We also identified contact sites in the other OXPHOS complexes (based on previously published structures; Iwata et al. 1998; Zhou et al. 2015; Fiedorczuk et al. 2016) to classify N-mt OXPHOS proteins as “contact” if they contacted at least one mt residue and to test whether *d*_N_/*d*_S_ values increased as the proportion of contact residues in a gene increased.

We identified mt and N-mt CIV sites under positive selection to test whether N-mt genes had more positively selected sites than mt genes, if positively selected N-mt sites were enriched for mt contacts, and if positively selected sites had N-mt changes that followed mt changes. For each CIV gene we used codeml in PAML to identify sites under positive selection in at least one of 12 diverse branches of the mammalian phylogeny and their descendent branches (e.g., one represented the last common ancestor of bats and their descendants, another that of primates and their descendants), based on a phylogeny of 48 species (i.e., model = 2, Nsites = 2; supplementary fig. S6, Supplementary Material online). In some analyses we considered all sites identified as possibly being under positive selection and in others we only considered sites that passed a Bayes Empirical Bayes analyses (*P* < 0.05, Yang et al. 2005).

From those data, we determined the substitution order of contact pairs (mt first or N-mt first) by identifying on which branch of the consensus tree (fig. 5) mt and N-mt substitutions occurred using custom R scripts. Because not all comparisons of branch substitutions make sense (e.g., branches within mutually exclusive monophyletic clades), we identified the unique root to tip path for each species and restricted the classification of “mt first” or “N-mt first” to branches only within those paths. For example, site 92 of N-mt gene *cox*4 contacts site 463 of mt gene *cox*1. If we observed a *cox4*_92 substitution on a branch closer to the tip of the tree and a *cox*1_463 substitution on a branch closer to the root of the tree, we would code this as an mt substitution occurring first, followed by an N-mt substitution. We restricted some analyses to contact pairs that contain N-mt genes that either did or did not show evidence of positive selection based on the analyses above. We also restricted these comparisons in some analyses to substitutions that were two branches apart or fewer—but not on the same branch—and within a monophyletic clade (i.e., “close” vs. “far” substitutions in fig. 5). This was based on the reasoning that compensatory substitutions might occur relatively quickly after deleterious ones (although the two branches’ timing represents different absolute times across the phylogeny). Some analyses were restricted to primate branches only, given previous results (Osada and Akashi 2012). Finally, we filtered our dataset to only those substitutions that were predicted to have functional consequences to protein function (following Levin and Mishmar 2017).

From these comparisons of substitution timing, we calculated the proportion of mt first and N-mt first substitutions. We then conducted chi-square tests to evaluate the prediction that N-mt substitutions should occur after mt substitutions if nuclear compensation is driving mitonuclear coevolution. We also performed fisher exact tests to calculate the odds that mt substitutions occurred first within a specific dataset compared to a broader dataset. Specifically, we asked if the odds of mt first substitutions were higher in the data where N-mt residues were under positive selection, when substitutions occurred on nearby branches, and in functional nodal mutations.

## Supplementary Material

msac233_Supplementary_Data

## Data Availability

The sequences, alignments, other data, and code used to complete this study are available at https://doi.org/10.6084/m9.figshare.19350533.v2
