# Supplementary material for: Genomic Signatures of Mitonuclear Coevolution in Mammals
Source: Mol Biol Evol. 2022 Oct 27;39(11):msac233. doi: 10.1093/molbev/msac233 (PMC9641969; doi:10.1093/molbev/msac233)
Supplement: msac233_Supplementary_Data [file msac233_supplementary_data.zip › Supplementary_results_tables.pdf]

Table S1. Summary of datasets compiled for analysis

Dataset	genes	Genome	ETS Complexes	mt interaction	mt-Contact or Non-contact
mt	13	mitochondrial	I,III,IV,V		
mt-CI	7	mitochondrial	I		
mt-CIII	1	mitochondrial	III		
mt-CIV	3	mitochondrial	IV		
mt-CV	2	mitochondrial	V		
N-mt	74	nuclear	all	yes	both
N-mt-CI	37	nuclear	I	yes	both
N-mt-CII	4	nuclear	II	no	no
N-mt-CIII	8	nuclear	III	yes	both
N-mt-CIV	13	nuclear	IV	yes	both
N-mt-CV	12	nuclear	V	yes	both
N-mt contact	42	nuclear	I,III,IV,V	yes	contact
N-mt non-contact	28	nuclear	I,III,IV,V	yes	non-contact
Glycolysis	9	nuclear	none	no	
Cell cycle	13	nuclear	none	no	
N-mrp	40	nuclear	none	yes	
Cytosolic-ribosomal	20	nuclear	none	no	
Random N orthologs 1	15	nuclear	none	no	
Random N orthologs 2	15	nuclear	none	no	

Table S2. Summary of ERC results under different scenarios

Scenario	Comparison	Correlation (r_s)
Original analysis	mt - Nmt	0.82
Primates removed	mt - Nmt	0.66
Account for phylogenetic signal	mt - Nmt	0.71
Original analysis	mt - Glycolysis	0.25
Primates removed	mt - Glycolysis	0.48
Account for phylogenetic signal	mt - Glycolysis	0.19
Original analysis	mt - Cell Cycle	0.24
Primates removed	mt - Cell Cycle	-0.08
Account for phylogenetic signal	mt - Cell Cycle	-0.13
Original analysis	mt - Nmrp	0.57
Primates removed	mt - Nmrp	0.22
Account for phylogenetic signal	mt - Nmrp	0.44
Original analysis	mt - Cytoribo	0.01
Primates removed	mt - Cytoribo	-0.11
Account for phylogenetic signal	mt - Cytoribo	0.15

Table S3. Summary of substitution timing results under different scenarios

Scenario	Criteria	mt first count	N-mt first count	mt first proportion	N-mt first proportion
Functional nodal mutations only	Overall	32	114	0.22	0.78
Original analysis	Overall	74	323	0.19	0.81
Functional nodal mutations only	Positive selection	2	2	0.5	0.5
Original analysis	Positive selection	9	42	0.18	0.82
Functional nodal mutations only	No selection	30	112	0.21	0.79
Original analysis	No selection	62	281	0.18	0.82
Functional nodal mutations only	Close	6	14	0.3	0.7
Original analysis	Close	12	25	0.32	0.68
Functional nodal mutations only	Far	26	85	0.23	0.77
Original analysis	Far	62	254	0.2	0.8
