# Supplementary figures and images for: Genomic Signatures of Mitonuclear Coevolution in Mammals
Source: Mol Biol Evol. 2022 Oct 27;39(11):msac233. doi: 10.1093/molbev/msac233 (PMC9641969; doi:10.1093/molbev/msac233)

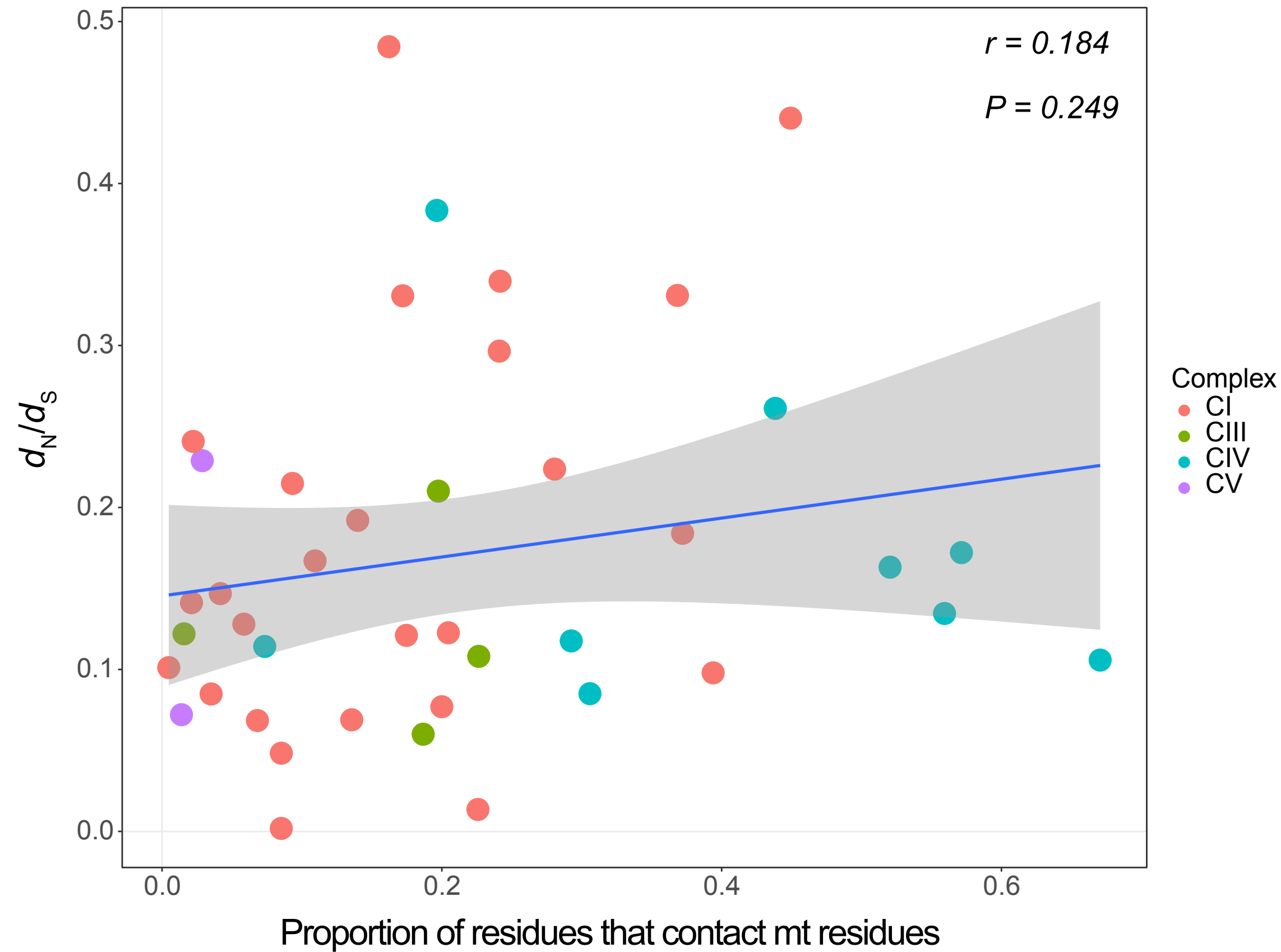

Supplement: msac233_Supplementary_Data [file msac233_supplementary_data.zip › FigS5_dNdS_correlation.pdf]

Proportion of sites under  
positive selection

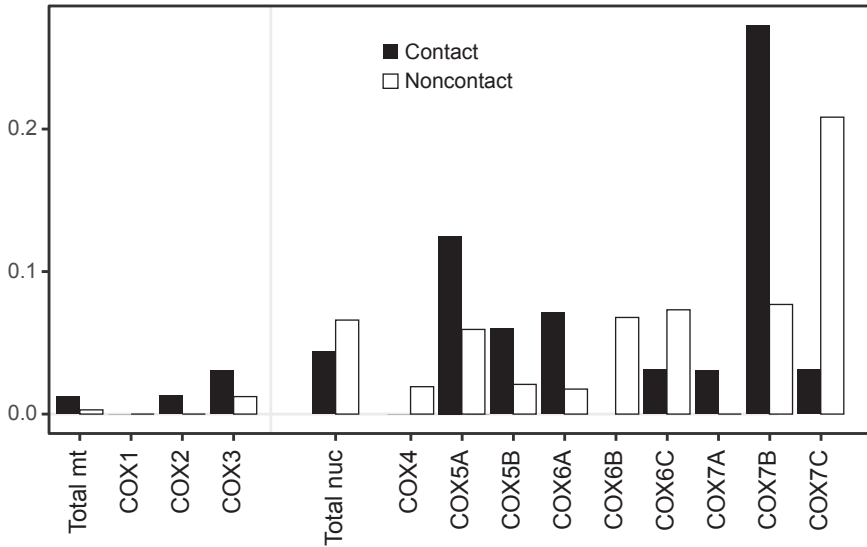

Supplement: msac233_Supplementary_Data [file msac233_supplementary_data.zip › FigS6_contactPosSelection_stringent.pdf]

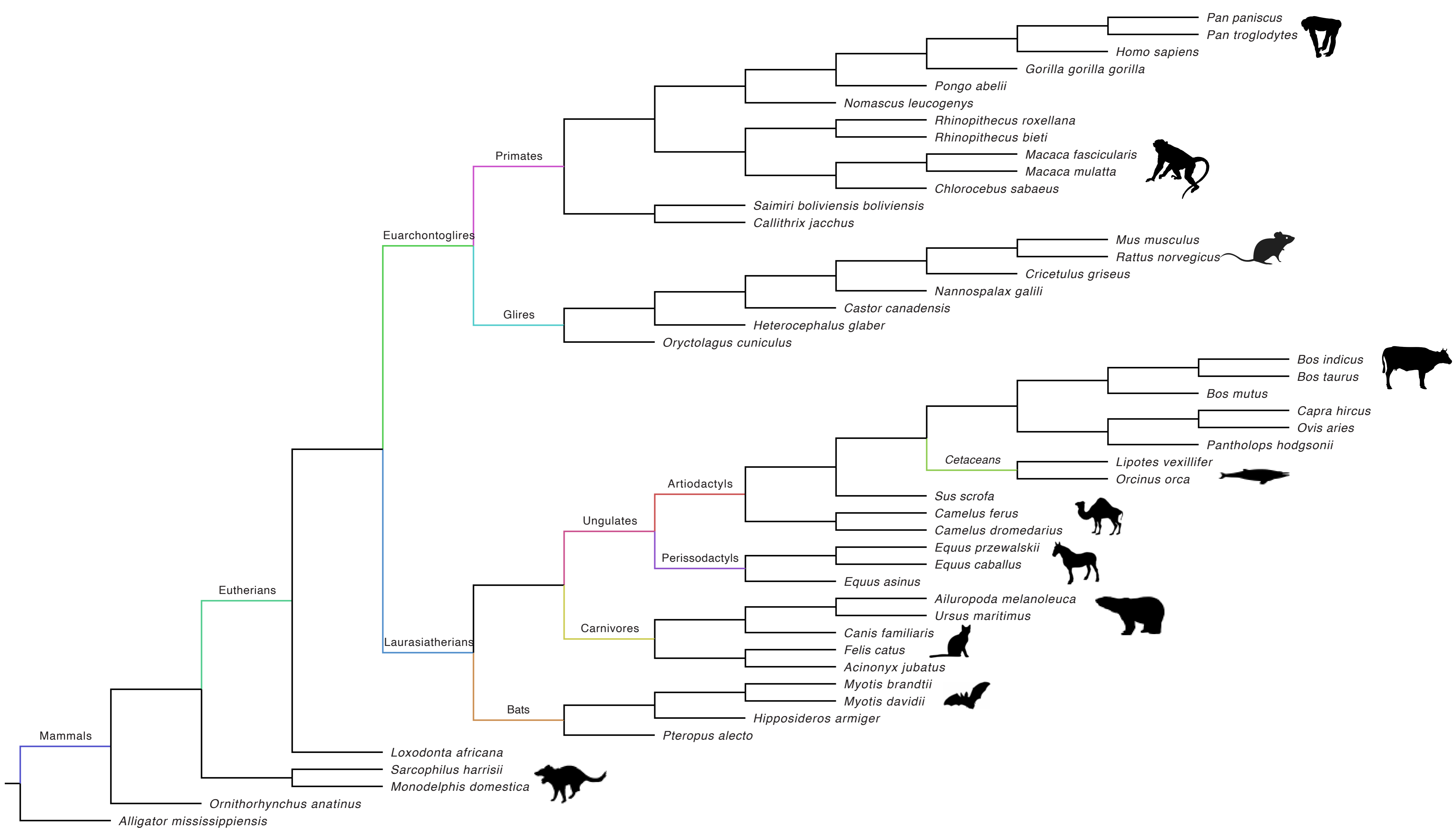

Supplement: msac233_Supplementary_Data [file msac233_supplementary_data.zip › FigS7_Phylogeny_wBranches.pdf]

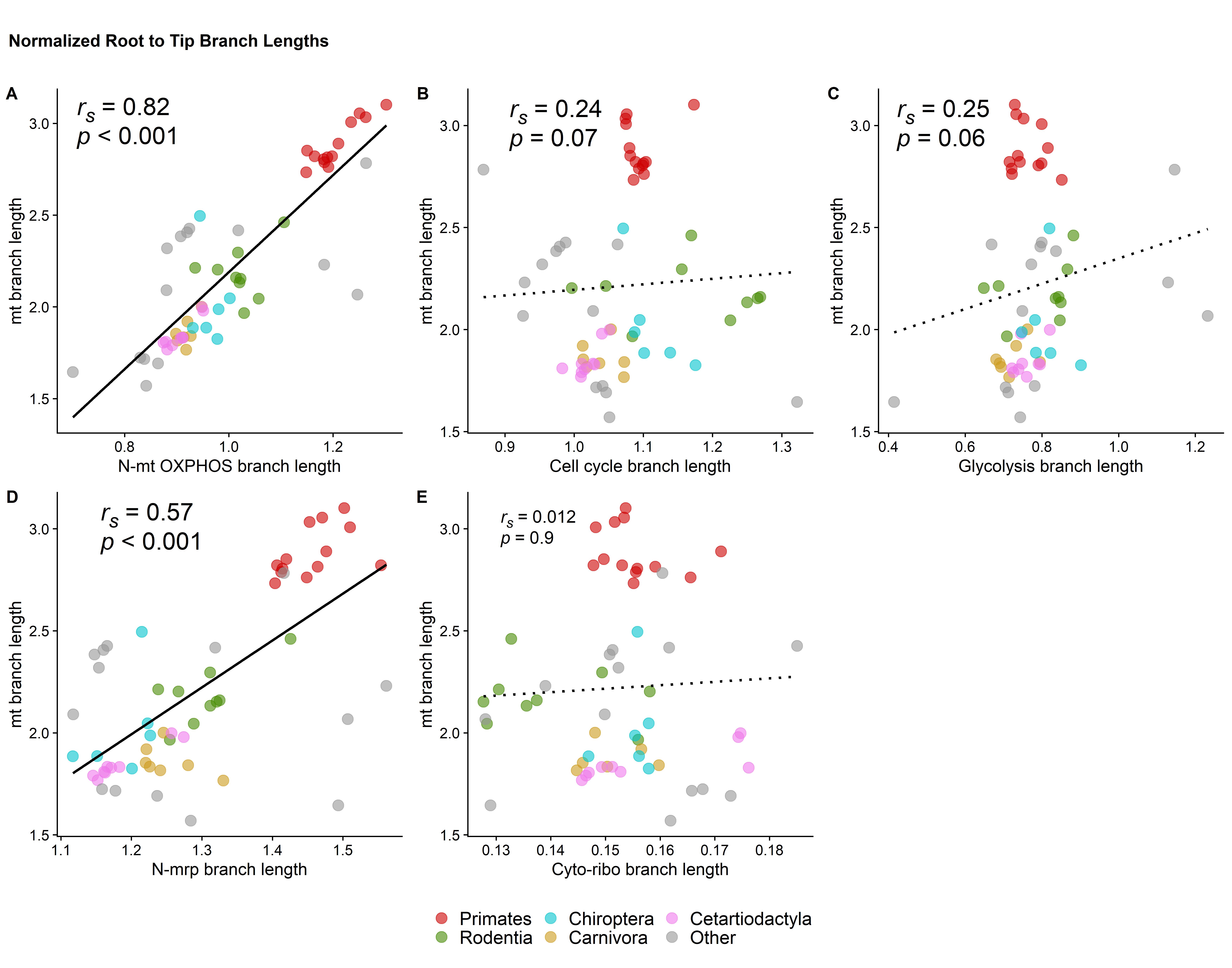

Supplement: msac233_Supplementary_Data [file msac233_supplementary_data.zip › Figure_S1_ERC_correlations.jpg]

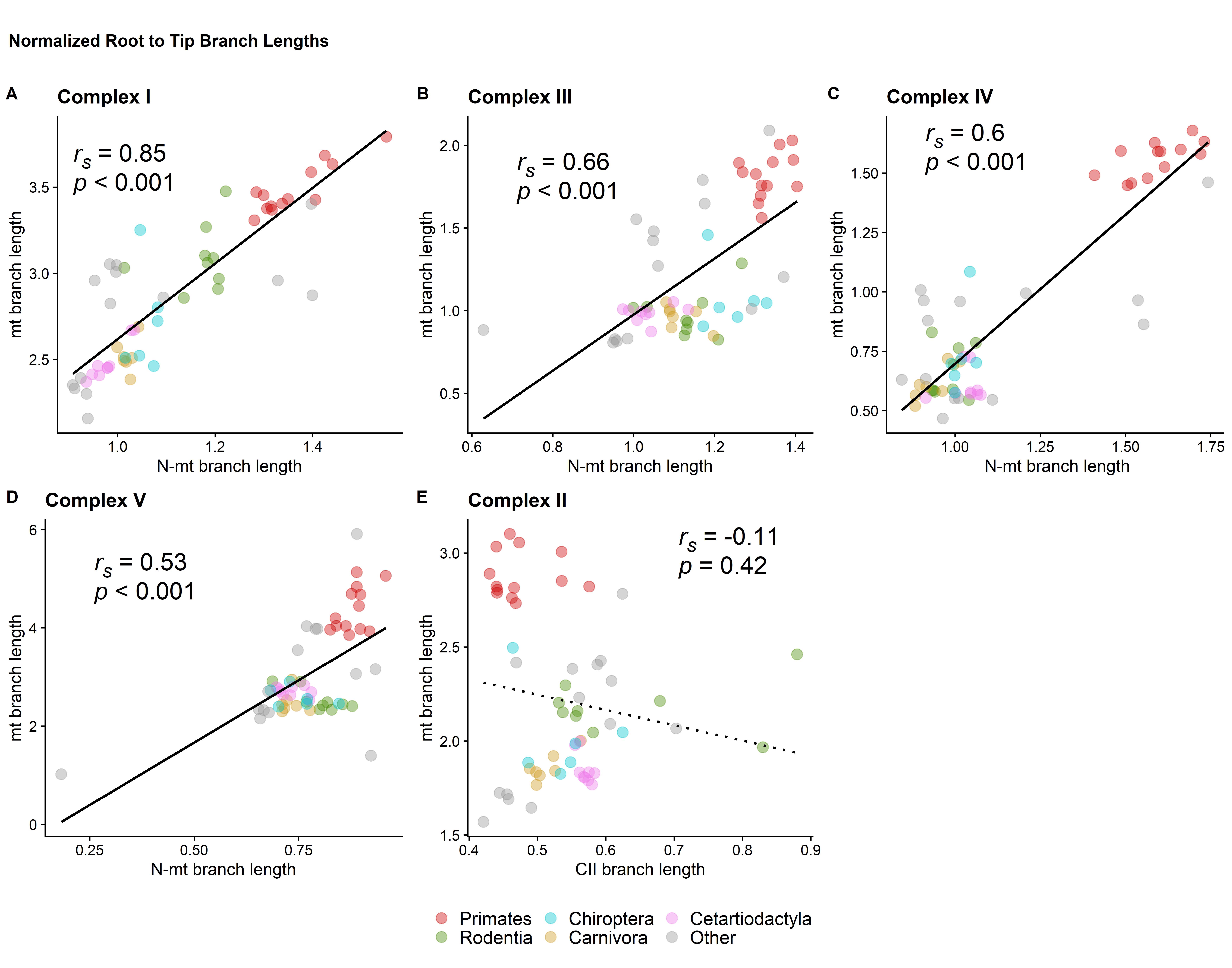

Supplement: msac233_Supplementary_Data [file msac233_supplementary_data.zip › Figure_S2_ETS_Complex_Corr.jpg]

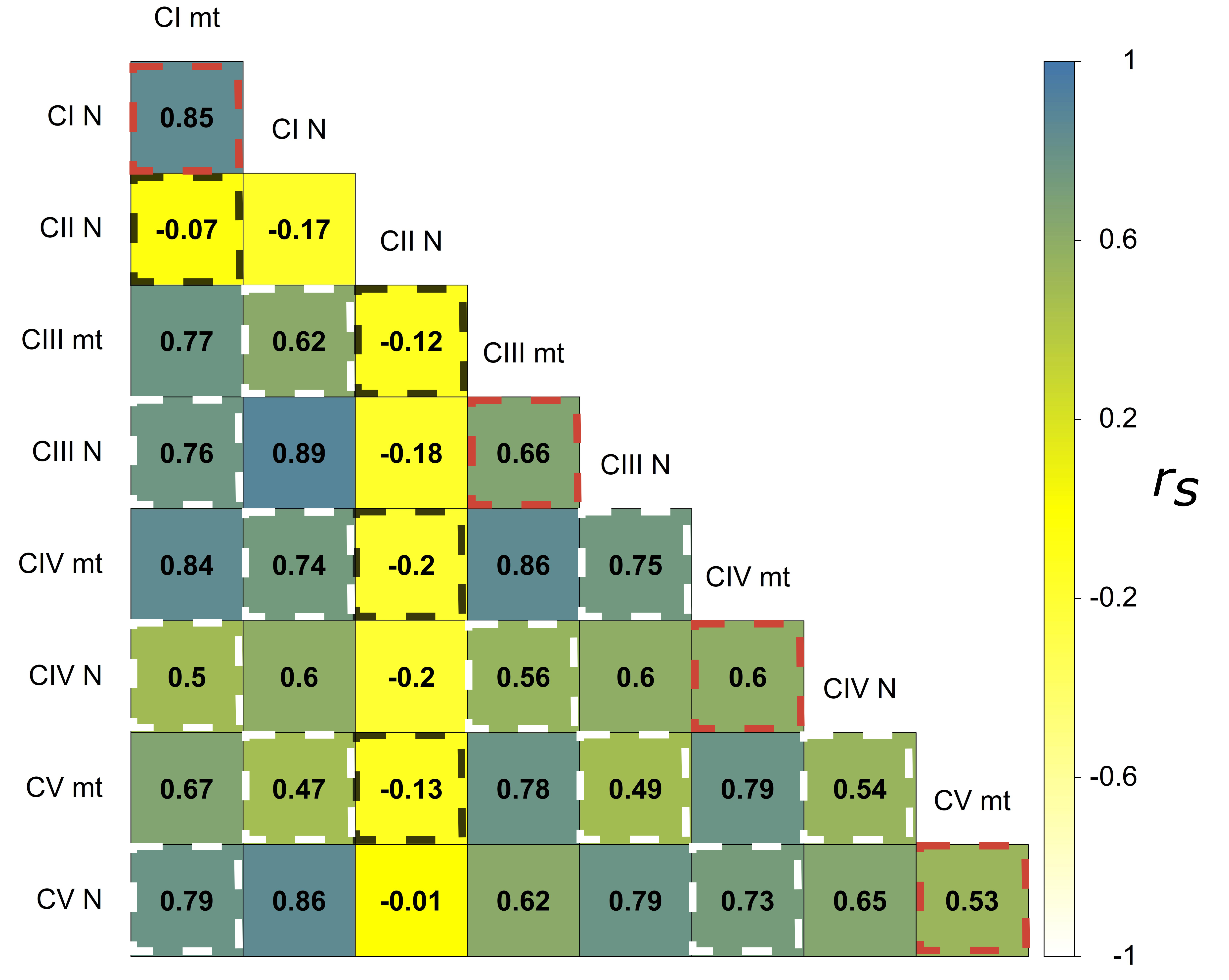

Supplement: msac233_Supplementary_Data [file msac233_supplementary_data.zip › Figure_S3_Correlation_Matrix.jpg]

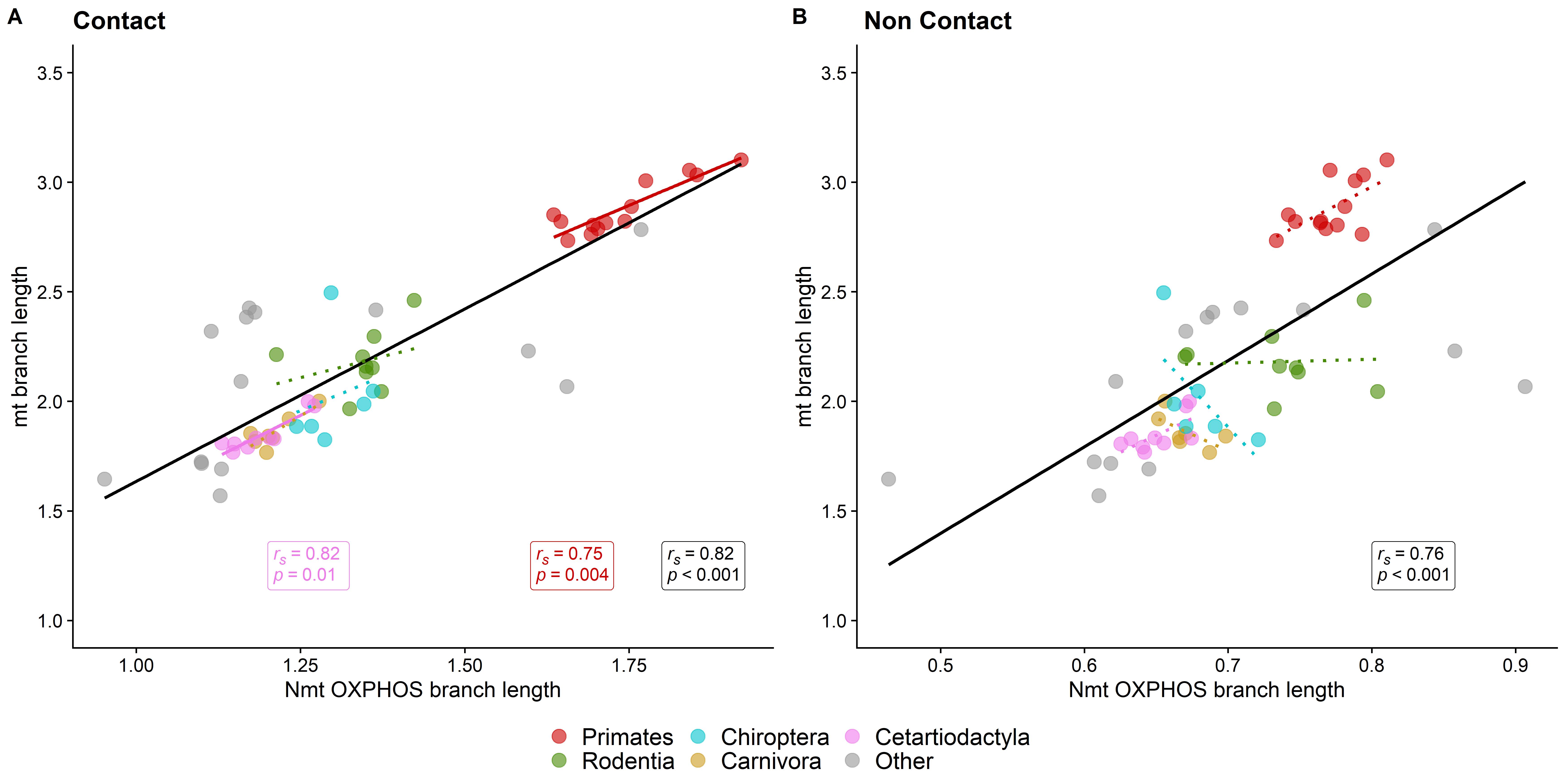

Supplement: msac233_Supplementary_Data [file msac233_supplementary_data.zip › Figure_S4_contact-non-corr.jpg]
